# Changes in blood glucose concentrations over time when administering intravenous insulin in post cardiac surgery in adult intensive care patients

**DOI:** 10.1186/2197-425X-3-S1-A189

**Published:** 2015-10-01

**Authors:** F Bass, N Hammond, S Bird, J Myburgh, S Finfer

**Affiliations:** Intensive Care Unit, Royal North Shore Hospital, Sydney, Australia; The George Institute for Global Health, Sydney, Australia; University of New South Wales, Sydney, Australia; Intensive Care Unit, St George Hospital, Sydney, Australia; The University of Sydney, Sydney, Australia

## Introduction

Patients in intensive care units (ICUs) are treated with intravenous (IV) insulin by infusions with the goal of maintaining blood glucose (BG) within a narrow target range. However, as BG is measured intermittently and how the time course of BG concentration changes after altering the insulin infusion rate is unknown. We used an intra-arterial Continuous Glucose Monitoring (CGM) system to study the time course of BG concentrations after changes in IV insulin infusion rates.

## Methods

This prospective, observational cohort study was conducted during Feb-April 2013 as part of a CGM product development study. Following local human research ethics approval we studied adult patients admitted to our ICU immediately after elective, cardiac surgery who received an IV insulin infusion as clinically indicated. Prior written consent was obtained from all participants prior to surgery. All BG concentrations were measured for up to 48hrs using a CGM system via the radial artery catheter. The CGM system recorded BG values every 10 seconds. Insulin (Actrapid) was infused at a concentration of I unit/5mls via a volumetric pump. We analysed BG values measured by the CGM system following a step-wise change in the insulin infusion rate for a maximum of 2 hours. Infusion rate changes analysed were (1 u/hr increase, 1 u/hr decrease, 2 u/hr increase,and 2 u/hr decrease).We excluded artefactual measurements that occurred due to blood draws or flushing though the arterial line. Linear interpolation was used to estimate excluded data.

## Results

**Figure 1:**
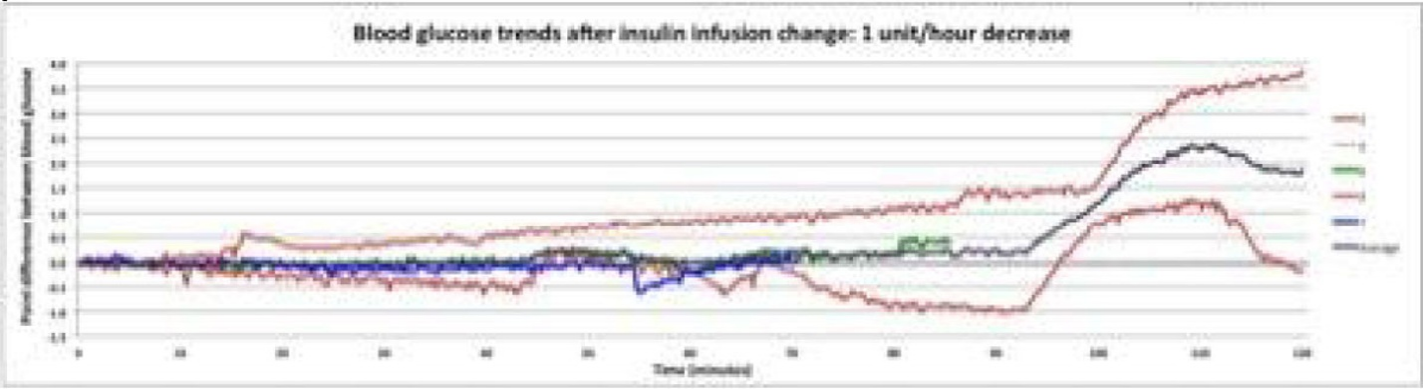
**Blood glucose trends after insulin infusion change.**

7 participants were treated with an IV insulin infusion during the recruitment period. Over the 2 hour time period the BG level of patients were variable. There were 5 episodes where the insulin infusion was decreased by 1 u/hr, BG remained relatively stable until 90 mins after which time there was a marked variability. In the group where insulin was increased by 2 u/hr there were 3 episodes where BG remained relatively stable, after 20 mins after which time obvious variability occurred. The group where insulin was decreased by 2 u/hr there were 3 episodes where there was no consistent pattern and variability in BG occurred within the first 30-40 mins (Figure 1).

## Conclusions

In patients studied immediately following cardiac surgery blood glucose concentrations following changes in an insulin infusion rate are highly variable. These data suggest that factors other than the insulin infusion rate determine BG concentration in this setting and that frequent checks of BG concentrations or CGM are needed to safely manage BG.

**Figure 2:**
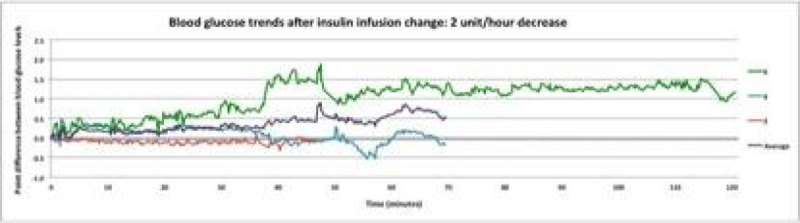
**BG trends after insulin infusion change:2u/hr decr.**

**Figure 3:**
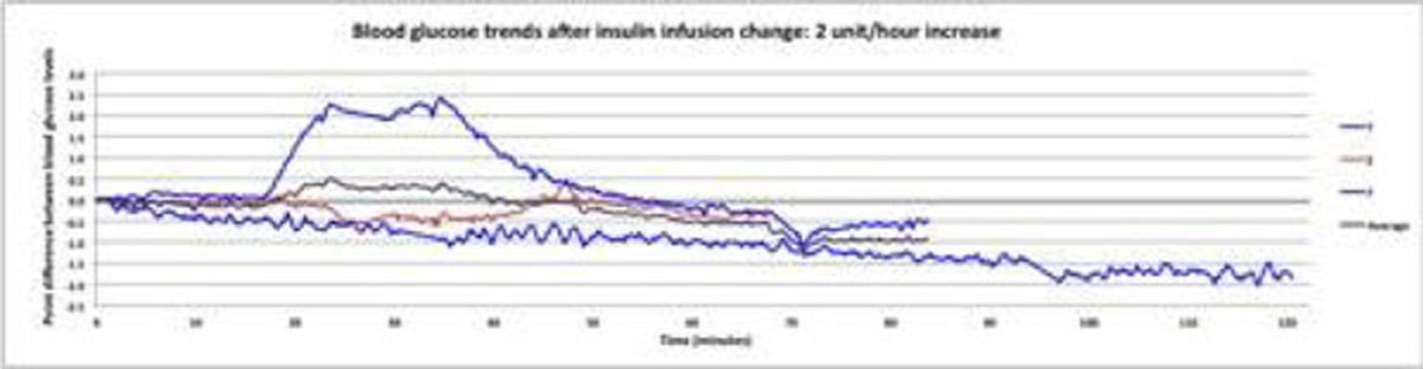
**BG treans after insulin infusion change:2u/hr incr.**

